# Socioeconomic inequality in child injury in Bangladesh – implication for developing countries

**DOI:** 10.1186/1475-9276-8-7

**Published:** 2009-03-23

**Authors:** Sheikh M Giashuddin, Aminur Rahman, Fazlur Rahman, Saidur Rahman Mashreky, Salim Mahmud Chowdhury, Michael Linnan, Shumona Shafinaz

**Affiliations:** 1 Department of Statistics, Jagannath University, Dhaka, Bangladesh; 2 Center for Injury Prevention and Research Bangladesh, Dhaka, Bangladesh; 3 The Alliance for Safe Children, Bangkok, Thailand; 4 UNICEF Bangladesh, Dhaka, Bangladesh

## Abstract

**Background:**

Child injury is an emerging public health issue in both developed and developing countries. It is the main cause of deaths and disabilities of children after infancy. The aim of this study was to investigate the socioeconomic inequality in injury related morbidity and mortality among 1–4 years children.

**Materials and methods:**

Data used for this study derived from Bangladesh Health and Injury Survey. A multistage cluster sampling technique was conducted for this survey. In this study quintiles of socioeconomic status were calculated on the basis of assets and wealth score by using principle component analysis. The numerical measures of inequality in mortality and morbidity were assessed by the concentration index.

**Results:**

The poorest-richest quintile ratio of mortality due to injury was 6.0 whereas this ratio was 5.6 and 5.5 for the infectious diseases and non-communicable diseases. The values of mortality concentration indices for child mortality due to infection, non-communicable diseases and injury causes were -0.40, -0.32 and -0.26 respectively. Among the morbidity concentration indices, injury showed significantly greater inequality. All the concentration indices revealed that there were significant inequalities among the groups. The logistic regression analysis indicated that poor children were 2.8 times more likelihood to suffer from injury mortality than rich children, taking into account all the other factors.

**Conclusion:**

Despite concentration indices used in this study, the analysis reflected the family's socioeconomic position in a Bangladesh context, showing a very strong statistical association with child mortality. Due to the existing socioeconomic situation in Bangladesh, the poor children were more vulnerable to injury occurrence.

## Background

In recent decades overall child mortality rates have decreased globally. In Bangladesh, infant mortality declined by 25 percent from 87 deaths per 1,000 live births to 65 deaths per 1,000 live births between the periods 1989–2004. Child and under five mortality declined 52 and 34 percent respectively over the same period. In the last five years, child mortality declined only 20 percent [1]. Various immunization and intervention programs played an important role in reducing the under five mortality. These programs are mainly focused on reducing infectious and non-communicable diseases. However, injury emerged as a leading cause of both morbidity and mortality for children. Studies from Bangladesh revealed that 21% deaths occurred among 1–4 year's children due to injury [1-3]. In infancy, the main causes of death were low birth weight, pneumonia and birth asphyxia, whereas after the infancy, the leading cause of child death is injury [1]. It is now a public health issue in both the developed and developing world [4,5]. The World Health Organization reported almost 6 million deaths due to injury [6]. It is also a major cause of disabilities and deaths of children [7,8]. The problems of road traffic accident, drowning, fall, and burn have been an unnoticed public health disaster. It is expected that injury will be the rival of communicable disease as a cause of ill health and death by the first decade of the new millennium [9]. Non-fatal injuries are the frequent cause of hospital admission and disabilities center admissions, and half of the hospital surgical beds are occupied by injury patients [10]. Within developing countries, poor people represented by pedestrians, passengers in buses and trucks, and cyclists suffer a higher burden of morbidity and mortality from traffic injuries [11]. Data from several populations suggest that socioeconomic disparities are strong predictors of childhood mortality [12-16]. Low maternal education, young age, and increased number of children were strong predictors of injury mortality rate for children 0 to 4 years of age [12]. Although the recent decline in child mortality in Bangladesh is remarkable, death from different causes other than infectious diseases and malnutrition remains an important component of child mortality. Child death due to injury especially drowning can be expected to be a problem in Bangladesh given the geographical features of the country [17]. Rivers are the most important geographical features in Bangladesh. It's been known that the out flow of water from Bangladesh is the third highest in the world, after the Amazon and the Congo systems. The Padma, Jamuna and the lower Meghna are the widest rivers. Some rivers are known by different names in various portions of their course. Throughout the country there are bils, haors and lakes that meet the need of irrigating water. Bangladesh has a tropical monsoon-type climate, with a hot and rainy summer and a dry winter. Most rains occur during the monsoon (June – September) and little in winter (November – February). Bangladesh is subject to devastating cyclones, originating over the Bay of Bengal, in the periods of April to May and September to November. Often accompanied by surging waves, these storms can cause great damage and loss of life.

Globally, poorer countries bear a disproportionate burden of injury morbidity and mortality. Numerous studies have demonstrated that lower socioeconomic classes have higher death rates than upper socioeconomic classes, and this difference has increased in the past decades [1,18,19]. However, very few studies have focused on the equity in child injury mortality and morbidity. The aim of this study was to investigate how the socioeconomic position associated with injury morbidity and mortality among 1–4 year-old children. Further, the aim was to explore what extent the risk factors could explain some of the socioeconomic inequity in injury mortality and injury morbidity of the children. We hypothesized mother's age, mother's education, number of living children, sex of child and household's income to represent different aspects of socioeconomic position and, therefore, to have an association with occurrence of injury mortality and injury morbidity.

## Materials and methods

### Data

The data used for this study was derived from Bangladesh Health and Injury Survey [20]. The aim of the survey was to investigate information on the cause of death and serious morbidity and to observe the patterns, characteristics of injury and overall risk factors and hazards for childhood injuries. The survey also investigated environmental risks and hazards around households. This study was conducted in Bangladesh between January and December 2003. It was a large cross sectional survey across the country. A multistage cluster sampling technique was conducted for this survey.

Administratively, Bangladesh is divided into 6 divisions. These in turn consist of 64 districts, 12 of them randomly selected. In each district, one upazila (sub-district) was randomly selected. In each upazila, two rural unions (the smallest administrative unit) were randomly selected. A total of 24 unions were selected for this study. Each rural union had roughly 20,000 populations. The district headquarters of the 12 selected districts constituted the urban areas. In the urban areas, *mohallas *(smallest administrative unit in urban areas) served as cluster and systematic sampling was done to achieve the required number of households. A sample of 132,000 households from both rural and urban areas in Bangladesh was selected for collecting information.

In the survey, respondents were asked to provide a complete history of each individual, including age, sex and occurrence of death (if any) in the households during the preceding two years before the survey. Any illnesses of the household's members which occurred 6 months prior to the survey were also recorded. For those who died, age and sex details were recorded. Verbal autopsy was made to diagnose the causes of death and illness with a specified structured questionnaire. Two independent panels of pediatricians checked the collected autopsy forms to assign the exact cause of death. All the causes were classified into three broad categories such as infection (due to the entry and development or multiplication of an infectious agent in the body, for example ARI/Pneumonia, Diarrhoea etc), non-communicable disease (diseases not capable of being directly or indirectly transmitted from person to person, for example Malnutrition, Birth asphyxia, Asthma etc.) and injury (physical damage due to the transfer of energy. Injury occurs when the amount of energy transfer exceeded the host organism's threshold tolerance. The type of energy can be mechanical, thermal, chemical, electrical, radiation or the absence of essentials such as oxygen or heat). To collect information on characteristics of injuries, separate forms were used for each mechanism of injury.

### Statistical analysis

**Figure 1:**
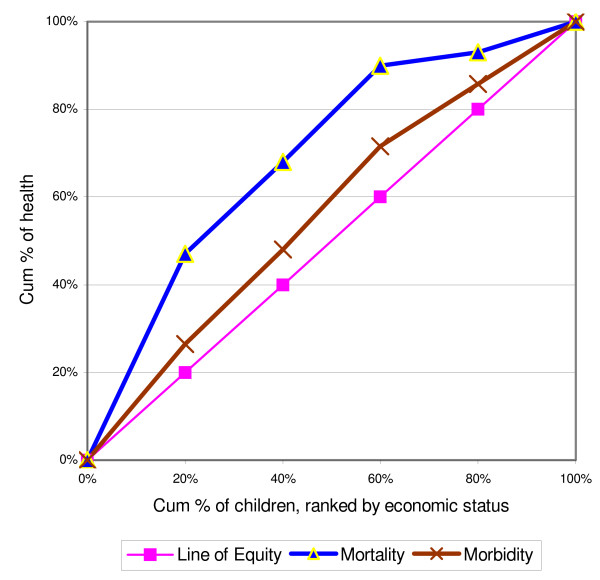
Mortality and morbidity concentration curve.

This study used a proxy measure of economic status of each household in terms of assets or wealth, (Poorest, Second, Middle, Fourth, Richest), rather than in terms of income or consumption. Information regarding the household items (i.e. television, radio, electricity, refrigerator or car), ownership of house, household's income (price of all commodities produce in a year from agriculture and other sources plus salaries of household's members if applicable) and cultivable land were assigned a weight or factor score generated through principle component analysis [19]. Principal component is a technique for extracting from a set of variables those few orthogonal linear combinations of the variables that capture the most common information successfully. Intuitively the first principal component of a set of variables is the linear index of all the variables that capture the largest amount of information that common to all of the variables. The resulting scores distributed normally with mean zero and standard deviation one. Each household was assigned a standard score for each asset. Standard household score was added up for each household, and each child was assigned the total household asset score for its household. Children were ranked according to their total scores and divided into five quintiles to understand health inequality. Inequalities by income in mortality and morbidity thereof are measured here using a concentration index and concentration curve [19]. To measure income related inequality in health we plot the concentration curve, which graphs the cumulative proportion of health against the cumulative proportion of population ranked by income (see Figure 1). It plots the cumulative proportion of deaths or illnesses (on the *y*-axis) against the cumulative proportion of children at risk (on the *x*-axis), ranked by income, beginning with the most disadvantaged child. If the curve coincides with the diagonal, all children, irrespective of their household income, enjoy the same mortality rates. The convention is that the index takes a negative value when the curve lies above the line of equality, indicating disproportionate concentration of the mortality among the poor, and a positive value when it lies below the line of equality. The further the curve lays from the diagonal, the greater the degree of inequality in mortality across quintiles of economic status. Concentration index is a generalization of the Gini coefficient. The numerical measure of inequality in mortality is measured by the *concentration index *denoted as C defined as twice the area between the concentration curve and the diagonal. The lowest value that C can take -1: this occurs when all the population's health is concentrated in the hands of most disadvantaged person. The maximum value of the index can take +1: this occurs when all the population's health is concentrated in the hands of least disadvantaged person. The concentration index estimates reported in the study calculated from grouped data using the following equation:

(1) C = 2/*μ*(Σ *f*_*t *_*μ*_*t *_*R*_*t*_)-1

Where, *μ *represents the mean of the particular health indicator *μ*_*t *_and *f*_*t *_respectively represent the value of health indicator and population share for the t^*th *^socio-economic group. R_t _is the relative rank of the t^*th *^socio-economic group, defined as , which indicates the cumulative proportion of population up to midpoint of each interval group.

Analysis of the effects of socio-economic and demographic factors on child injury mortality and morbidity was based on the estimation of a primary model. The model examined the effects of maternal and other socio-demographic characteristics on the likelihood of the child being injured. In the analysis of the model, death or illness due to injury measured as dichotomous variable coded 1 if the injured prior to the date of interview and 0 otherwise. Logistic regression model used for the analysis. The coefficient in the analysis represented increase or decrease in the log odds of being occurrence of event (versus not occurrence) associated with a unit or category change in an independent variable.

### Limitation and strength

It was a large sample study. To ensure the sufficient number of deaths, the recall period was two years in the survey, but deaths of one-year recall period were used for this study. Data collected using verbal autopsy or verbal diagnosis checklist, which might be some misclassification of causes of death determined by non-physician data collectors. Socioeconomic information was collected on the report of the respondent; so, there would be a possibility of underreporting of socioeconomic data. The analysis was mainly based on the proportion of occurring injury. Due to lack of sufficient information of the denominator, rate could not be measured. Asset score measured in this study was consistent with the Bangladesh Demographic Health Survey (BDHS). Values of some quintiles were 0 (Zero) for specific causes of injury, so the concentration index was not measured.

## Results

Table 1 shows the descriptive measures of each household's durable goods. Results of this study show that about 27 percent of households own a television, 32 percent own either a radio or a tape recorder, 3 percent had a refrigerator. Almost 91 percent of Bangladesh's upper class households had televisions and only 2.7 percent middle class households had a television. Only the 15 percent of the rich quintiles' household had ownership of a refrigerator. About one-third (31 percent) of the households of the study sample had an electricity connection. About 80 percent of households had their own homestead. Only 39.7 percent of the households possessed cultivable land more than or equal to 50 decimal (i.e. half acre). In the survey, respondents were asked their gross monthly income. Almost 50 percent of the household respondent mentioned that they earned more than 3000 BDT (Bangladeshi Taka) i.e. 50 USD.

**Table 1:** Percentage of household asset and economic characteristics by each quintile

	Poorest	Second	Middle	Fourth	Richest	Total
Possession of television	0.0	0.0	2.7	46.8	90.5	26.8
Possession of radio/tape-recorder	0.0	0.0	26.5	56.9	80.4	31.8
Possession of motor-cycle	0.0	0.0	2.0	10.0	88.0	1.6
Possession refrigerator	0.0	0.0	0.0	0.6	14.9	2.8
Electricity connection	0.0	0.0	22.5	35.9	84.6	31.2
Land> = 50 decimal	0.0	52.1	54.7	59.6	42.2	39.7
Income>3000 BDT	0.0	36.5	55.4	77.0	95.7	51.9
Ownership of House	94.2	92.5	87.5	75.9	41.2	80.4
Rent House	0.0	0.0	6.4	19.4	56.3	15.5

The ratio of mortality and morbidity proportion by different causes and by each quintile is given in Table 2. The first, third and fifth quintiles of assets index referred as low, middle and high economics status. Due to shortage of space, two intermediate (second and fourth) quintiles are not shown in the Table. The overall mortality of 1–4 year children was consequently highest for those living in the poorest families (first quintile). For example, a poor-rich ratio 5.7 for children implies that child mortality in the poorest quintile was about 5.7 times more than that of the richest quintile. The results of this study revealed that injury related mortality and morbidity are much more among the most disadvantaged group of Bangladeshi children than infectious and non-communicable diseases. The poorest-richest quintiles ratio due to injury mortality was 6.0 whereas this ratio was 5.6 and 5.5 for the infectious and non-communicable diseases. Similarly, the ratio of poorest-richest quintile in terms of illness, had the highest injury ratio. The ratio of injury related illness of 1–4 years children between poor and rich was 1.7 whereas infectious and non-communicable disease was 1.2 and 1.6 respectively.

**Table 2:** Inequality in specific cause of mortality and morbidity by quintile

Category	Mortality							Morbidity						
	
	Q_1_	Q_3_	Q_5_	Q_1_/Q_5_	CI	SE (CI)	t-value	Q_1_	Q_3_	Q_5_	Q_1_/Q_5_	CI	SE (CI)	t-value
***Broad category***														
Infection	43.8	18.0	7.9	5.6	-.40***	0.08	-5.0	21.8	17.8	18.3	1.2	-.03	0.02	-1.5
NCD	39.3	21.4	7.1	5.5	-.32**	0.13	-3.1	22.7	21.0	14.3	1.6	-.08**	0.04	-2.2
Injury	40.0	21.7	6.7	6.0	-.26**	0.06	-3.2	23.9	22.4	14.0	1.7	-.09**	0.04	-2.2
***Leading causes***														
Pneumonia	50.0	11.1	8.3	6.0	-.34***	0.07	-5.2	20.4	20.3	18.8	1.1	0.0	0.02	0.1
Diarrhoea	50.0	10.0	10.0	5.0	-.33**	0.11	-3.0	29.0	14.5	13.5	2.1	-.13**	0.05	-2.3
Drowning	39.6	22.6	5.7	7.0	-.28**	0.11	-2.6	31.3	22.7	8.2	3.8	-.21**	0.08	-2.7
Total	41.8	19.8	7.3	5.7	-.32**	0.07	-4.2	22.5	19.3	16.8	1.4	-.05**	0.02	-2.6

NCD = Non-communicable Disease; Q_1 _= Poorest quintile; Q_3 _= Middle quintile; Q_5 _= Richest quintile; CI = Concentration Index; SE = Standard Error; *<.10;**P < .05; ***P < .01

The values of *Concentration Index (CI) *for child mortality for the infectious, non-communicable diseases and injury causes are -0.40, -0.32 and -0.26 respectively. All are significantly different from zero at conventional levels (the *t*-value are -5.0, -3.1 and -3.2 respectively). The absolute value of the CI of infectious disease 0.40 indicated there were higher degree inequalities in mortality. Similarly, among the morbidity concentration indices injury showed the significantly greater inequality.

**Figure 2:**
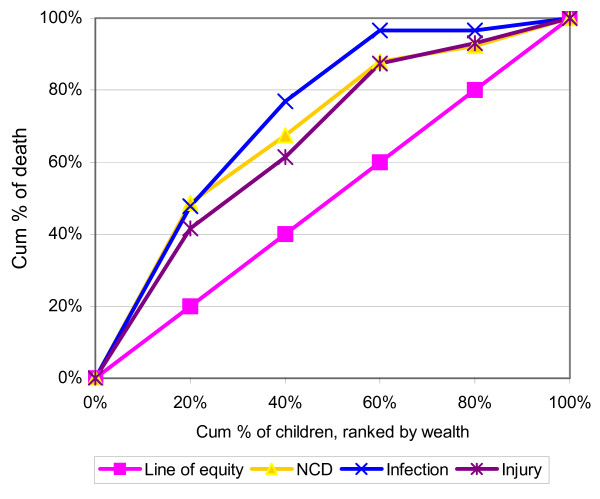
Mortality concentration curve by causes of death.

In Figure 1, the mortality concentration curve indicated the greater degree of inequality than the morbidity concentration curve. The absolute values of CI for mortality and morbidity are 0.32 and 0.05. Both are significantly different from zero (p < .05). The mortality concentration curves for deaths due to all causes lies above the line of equity (Figure 2). All the curves indicated that child mortality favored the better off. And the curve labeled infectious diseases lies everywhere further from the diagonal than the curve for non-communicable diseases and injuries. The curve for infectious diseases dominated that of non-communicable diseases and injuries. Although the infectious labeled curve was dominating, still a greater degree of inequality appeared in mortality among the income groups for the non-communicable diseases and injuries labeled curves.

The study found that pneumonia and diarrhoea were the top two leading causes of death of children between age 1 and 4. The study also found drowning was the third leading cause of childhood death. The mortality and morbidity inequality was highest for cause due to drowning (Table 2). The poor-rich ratios for drowning, diarrhoea and pneumonia was 7, 5 and 6 respectively. Concentration indices and its corresponding t-value reveal that there were significant inequalities among the income groups for these three leading cause of mortality. It is also found that cause of pneumonia had the greater degree of inequality.

In case of leading causes of morbidity, the poor-rich ratio for diarrhoea and pneumonia were 2.1 and 1.1 respectively. Drowning was another cause of childhood morbidity as well as mortality and the ratio was 3.8. Concentration indices for pneumonia, Diarrhoea and drowning were 0 (zero), -0.13 and -0.21. Significant inequality appears for the diarrhoeal and drowning causes of morbidity among the children (Table 2). Among the children, burn and unintentional poisoning injuries morbidity were 3.2 and 1.5 time more for most disadvantaged than the better off (Table 3). Electrocution was another cause of childhood morbidity as well as mortality.

**Table 3:** Inequality in specific causes of injury morbidity

Causes	Q_1_	Q_3_	Q_5_	Q_1_/Q_5_
RTA	15.2	20.7	23.9	0.6
Cut Injury	25.0	8.1	16.9	1.5
Burn	29.5	24.5	9.9	3.0
Drowning	31.3	22.7	8.2	3.8
Poison	23.1	23.1	15.4	1.5
Machine	23.1	46.2	7.7	3.0
Electrocution	21.6	29.4	2.0	11.0
Others	35.7	21.4	21.4	1.7

The logistic regression analyses are provided in Table 4. Analysis revealed that female children had a significantly higher likelihood of injury death compared to male children. The analysis also revealed that the households with many living children had a greater chance of experiencing a child injury death in the family. Children of poor families were 2.8-times more likely to suffer from injury mortality, taking into account all other factors (Table 4). It was also observed that the likelihood of morbidity due to injury was 1.3 times higher among children of poor families as compared to the children of rich families. Again, taking into account all factors, the occurrence of injury morbidity increased with the child's age. The likelihood of injury morbidity was significantly lower among the females as compared to the males. Children of older-age mothers were 20 percent less likely to have injury morbidity than the children with younger-age (<25 year) mothers. The odds ratio for experiencing an injury related morbidity for the poorest quintiles compared to the richest was 1.3 (95% CI = 1.04–1.62). However, the odd ratio for experiencing an injury related death for poorest quintiles compared to the richest was 2.8 (95% CI = 1.1–7.9).

**Table 4:** Multiple logistic regression model for risk of injury mortality and morbidity

Characteristics		Mortality		Morbidity	
		OR	95% CI	OR	95% CI
		
Child age in year		0.9	.68–1.1	1.6**	1.5–1.6
Child's Sex	Male	1.0		1.0	
	Female	1.6*	.95–2.7	0.85**	0.8–0.9
Economic status	Richest	1.0		1.00	
	Poorest	2.8**	1.1–7.9	1.30**	1.0–1.6
Mother's age	<25 years	1.0		1.0	
	> = 25 years	0.9	0.4–2.0	0.80*	0.7–0.9
Mother's Education	No education	1.21	0.64–3.7	1.27	0.73–2.70
	Primary	1.32	0.4–2.90	1.12	0.56–3.10
	Secondary+	1.00		1.00	
No of living children	1	1.00		1.00	
	2	1.30	0.4–3.6	1.10	0.9–1.3
	3	1.56	0.4–5.0	1.21	0.9–1.5
	4+	4.14**	1.4–11.8	1.15	1.0–1.4
Constant		-5.71		-2.27	
Model chi-square		41.3		322.8	
-2loglikelihood		610.9		6676.6	

OR = Odd ratio; *<0.10; **P < 0.05; ***P < 0.01

## Discussion

This study investigated the inequality in mortality and morbidity for one to four year old children, especially by cause of injury, among socio-economic groups. In this study, quintiles were used to measure disparities in mortality by the economic status in terms of household's goods that were developed by World Bank [19]. The concentration indices confirmed that injury related death was higher among the poor children as compared to rich children. The study found that inequalities also appeared in mortality due to infectious and non-communicable diseases. The children of poorest families suffered more in injury morbidity than the children of rich families. Income inequality was significantly visible in both leading causes of mortality and morbidity. Greater degree inequality exists in drowning related injuries among 1–4 year old children. The multivariate logistic regression analysis substantiated that female injury mortality was higher but the illness became lower than males. These might be the cause of gender discrimination. In most population, male mortality is higher than female mortality at almost all ages. In south Asia, however, female mortality is higher than male mortality at many ages especially during the childhood periods [21]. Excess female mortality during childhood in Bangladesh and other south Asian countries is believed to result from son preference, which leads to differential treatment of sons and daughters in terms of foods allocation, prevention of disease and accidents, and treatment of illness [22]. In south Asia different studies revealed the evidence of sons' preference and discrimination in caring for son and daughters and discrimination against girl children that leads to higher mortality rates [23,24].

The association of injury mortality and maternal education were statistically significant in the bivariate analysis. Maternal education was not statistically associated with child mortality in multivariate analysis although it is known as the main determinant of child survival in developing countries [25-28]. However, controlling for the economic variables and place of mothers residence in the multivariate analysis removed the statistical significance. This may indicate that this relationship is mediated by those variables. In recent years, other studies have found weak correlation between maternal education and child mortality in sub-Saharan Africa compared with results of other Third World regions [29,30]. There have been suggestions that the weak relation between maternal education and child injury mortality may be related to weaker health infrastructure in South Asian countries may in some way inhibit the ability of more educated women to take advantage of their human capital in the health environment.

The results of this study revealed significant association between socioeconomic inequality and incidence of injuries among children after adjustment of other factors. The study also stated that wealth distribution affected almost similar pattern on child mortality due to infectious, non-communicable diseases and injury. This analysis demonstrated that after infancy age is an important mediator of the relationship between economic inequality and injury mortality and morbidity.

Under the target for Millennium Development Goals (MDG) 4, Bangladesh is to reduce under-five mortality from 151 deaths per thousand live births in 1990 to 50 in 2015. Although there has been a decline among both infant and under five mortality, they are still considered to be very high compared to many developing countries. During the period 1991 to 2001, under-five mortality declined average 4.1 percent per year, which is below the required annual 4.3 percent, but from 1997 to 2001 the decline is only 1.6 percent per year [1]. A large part of these infant deaths are contributed by pneumonia and diarrhoea among children. Due to decreased infant mortality, the under-five mortality had decreased but child mortality remained constant since 1997. However, the third leading cause of under-five mortality and first leading cause of child mortality is drowning [1,20].

Findings from the other studies confirm that causes of injuries and the pattern of incidence by age and sex are more or less similar in developing countries and most of the injury incidence (i.e. cut, burn, falls, poisoning and drowning) of younger ages took places at the premises of home [31-33]. This study goes part of way towards highlighting the extent of ill health due to injury among poor children in developing countries. Based on the findings of this study, a direction of further research on inequity and child injury can be drawn.

### Conclusion and policy implication

According to the results it is concluded that the children of poorest families suffered more in injury morbidity and mortality than the children of rich families. Due to existing socioeconomic situation in Bangladesh, the poor children were more vulnerable to injury occurrence. The percentage of injured was much higher in the poor group as compare to rich group. Injury causes an extra burden to the poorest family as it results more disabilities than infectious and non-communicable diseases. So, there is an urgent need to develop programs to prevent injuries in low-income countries like Bangladesh. It is also important to reduce child injury instantly specially among 1–4 years children to achieve MDG 4 by 2015. In the health sector existing program should be given priorities for injury prevention efforts as well as vulnerable group.

## Competing interests

The authors declare that they have no competing interests.

## Authors' contributions

MSG participated in the planning of the study, performed the statistical analysis and drafted the manuscript. AR, FR, SRM, SMC, ML and SS participated in the planning of the study questions and analysis and were actively involved in reporting results and conclusion. All authors read and approved the final manuscript.
